# Ionic Liquids as Biocompatible Antibacterial Agents: A Case Study on Structure-Related Bioactivity on *Escherichia coli*

**DOI:** 10.1021/acsabm.2c00615

**Published:** 2022-10-19

**Authors:** Margarida M. Fernandes, Estela O. Carvalho, Daniela M. Correia, José M.S.S. Esperança, Jorge Padrão, Kristina Ivanova, Javier Hoyo, Tzanko Tzanov, Senentxu Lanceros-Mendez

**Affiliations:** † Centre of Physics, University of Minho, Braga4710-057, Portugal; ‡ Centre of Chemistry, University of Trás-os-Montes e Alto Douro, 5001-801Vila Real, Portugal; § LAQV, REQUIMTE, Departamento de Química, Faculdade de Ciências e Tecnologia, Universidade Nova de Lisboa, 2829-516Caparica, Portugal; ∥ Centre for Textile Science and Technology, University of Minho, Campus de Azurém, Guimarães4800-058, Portugal; ⊥ Grup de Biotecnologia Molecular i Industrial, Department of Chemical Engineering, Universitat Politècnica de Catalunya, 08222Terrassa, Spain; # BCMaterials, Basque Center for Materials, Applications and Nanostructures, UPV/EHU Science Park, 48940Leioa, Spain; ∇ Ikerbasque, Basque Foundation for Science, 48009Bilbao, Spain

**Keywords:** ionic liquids, antimicrobial, biocompatible, surface activity, Escherichia coli

## Abstract

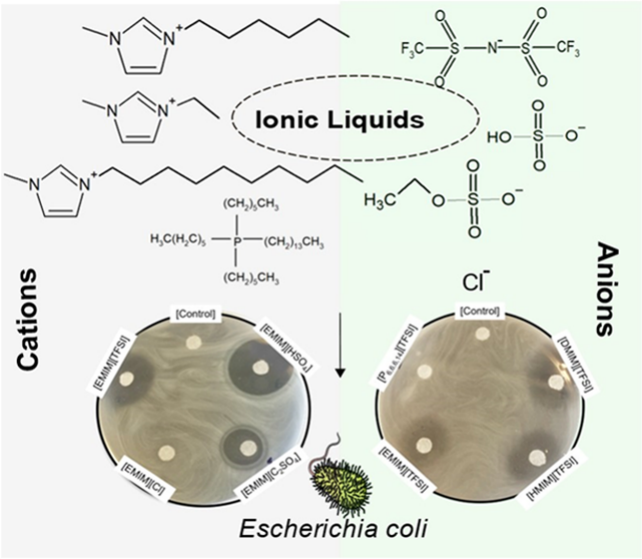

The potential of ionic liquids (ILs) to be used as antimicrobial agents for biomedical applications has been hindered by the fact that most of them are cytotoxic toward mammalian cells. Understanding the mechanism of bacterial and mammalian cellular damage of ILs is key to their safety design. In this work, we evaluate the antimicrobial activity and mode of action of several ILs with varying anions and cations toward the clinically relevant Gram-negative *Escherichia coli*. Langmuir monolayer technique was used to evaluate if the IL’s mode of action was related to the bacterial cell membrane interaction for an effective *E. coli* killing. 1-Decyl-3-methylimidazolium bis(trifluoromethylsulfonyl) imide [DMIM][TFSI] and trihexyltetradecyl phosphonium bis(trifluoromethylsulfonyl) imide [P_6,6,6,14_][TFSI] were surface-active and induced bacterial cell lysis, through a membrane-disruption phenomenon on bacteria, in a mechanism that was clearly related to the long alkyl chains of the cation. 1-Ethyl-3-methylimidazolium hydrogen sulfate [EMIM][HSO_4_] was highly antimicrobial toward *E. coli* and found suitable for biological applications since it was harmless to mammalian cells at most of the tested concentrations. The results suggest that the imidazolium cation of the ILs is mostly responsible not only for their antimicrobial activity but also for their cytotoxicity, and the inclusion of different anions may tailor the ILs’ biocompatibility without losing the capacity to kill bacteria, as is the case of [EMIM][HSO_4_]. Importantly, this IL was found to be highly antimicrobial even when incorporated in a polymeric matrix.

## 1 Introduction

Ionic liquids (ILs), which are composed of an organic cation and an inorganic or organic anion, are attracting significant research interest due to their versatility for an increasing number of technological applications.^1^ They are considered “green solvents” since their liquid state is not due to the presence of a solvent but rather due to the combination of a cation and anion, which determine the ILs’ polar and hydrophobic properties.^2,3^ Properties such as viscosity,^4,5^ melting temperature below room temperature,^6^ and high surface activity^7^ are relevant for developing biomedical applications. Nevertheless, the high surface activity may be the reason for the cytotoxicity of some ILs toward biological systems.^3,8,9^ While this is a concern for eukaryotic cells, it has been considered an interesting and important property in microbiology.^10^ ILs have been reported to have the ability to interact with microbial cell walls in a mechanism that involves their aggregation into the membrane components, disrupting the cell membrane integrity^11^ and showing their potential in antimicrobial applications.^12−14^ Moreover, ILs present specific chemical structures, which could be used for modifying the structure of antimicrobial agents/antibiotics to synergistically increase their efficacy through the introduction of tailored ILs’ cations or anions. In fact, introducing new molecules such as heteroatoms,^15^ chemical functionalities,^16^ and aromatic structures^17^ onto antibiotics is a strategy that has been used to increase their antimicrobial performances toward otherwise nonsusceptible bacteria.

Cationic compounds, including ammonium, imidazolium, pyridinium, and phosphonium, commonly found as cations in IL composition, have been reported to possess biocidal properties against a broad spectrum of bacteria^18^ and are widely used as disinfectants and cleaning agents in food and pharmaceutical industries and hospitals.^19^ This is why most of the studies with ILs for antimicrobial purposes report the cation as the reason for their antimicrobial activity and are often combined with antimicrobial inert anions such as bromide, chloride, and iodide.^4,18,20−22^ Changing the anions to antimicrobial active compounds may be the key to obtaining an improved antimicrobial agent. Despite this, the effect of the anions on the IL antimicrobial properties has been scarcely studied.

Some imidazolium-based ILs have already been proven to possess potent antibacterial activity, but the most suitable combination of cations and anions, including the effect of different cations, their chain length, and anion, and the mechanism of action, in combination with the balance between the toxicity toward bacterial cells and mammalian cells, have been scarcely highlighted.

This work reports on the antibacterial activity of different ILs against *Escherichia coli* and systematically investigates the effect of the cation, anion, and the size of the cation hydrocarbon chain on their antibacterial activity. Their membrane-disruption capacity has been further addressed using the Langmuir monolayer technique. *E. coli* is one of the predominant inhabitants of the human gastrointestinal tract, easily causing nosocomial infections in clinical settings. Specific pathogenic *E. coli* strains may express a variety of adhesins and easily colonize indwelling medical devices causing difficult-to-treat infections;^23^ thus, novel antimicrobial agents able to eradicate or prevent the spread of this specific bacterial strain are needed. The present work also allows us to define the proper concentration for the use of ILs in biological applications, i.e., a concentration that eradicates *E. coli* but is harmless to mammalian cells, and [EMIM][HSO_4_] as the most promising IL, which is also able to inhibit the bacterial growth when incorporated in a polymeric matrix.

## 2 Materials and Methods

### 2.1 Materials

The used ILs were commercially availed from Iolitec (purities >98%), except for trihexyltetradecyl phosphonium bis(trifluoromethylsulfonyl)imide, which was synthesized in-house by a metathesis reaction of [(C_6_H_13_)_3_P(C_14_H_29_)][Cl] with [Li][TFSI],^24^ following the procedures described in ref (25). The chemical structures of the ILs are presented in Table 1, together with the indication of the varying IL components. Highlighted in gray is the representation of ILs with the same 1-ethyl-3-methylimidazolium cation, changing the anions, and highlighted in green is the representation of ILs with the same bis(trifluoromethylsulfonyl)imide anion, changing the length of the alkyl chain linked to the imidazolium cation. All ILs were vacuumed (0.1 Pa) at 60 °C for at least 1 day prior to use.

**Table 1:**
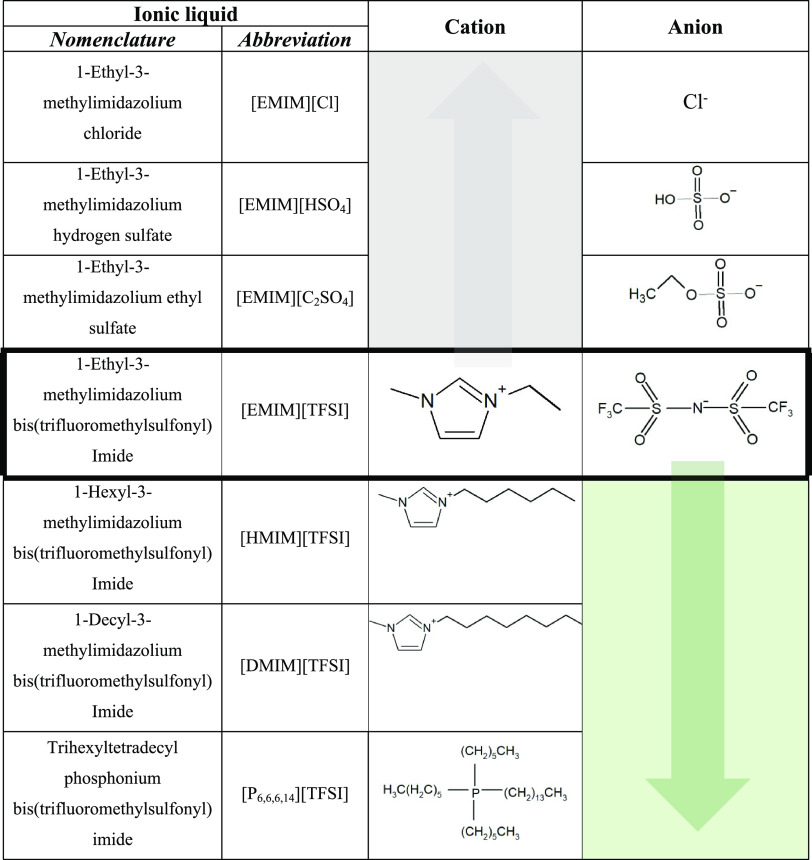
Ionic Liquid Nomenclature, Cation and Anion Chemical Structures, and Graphical Representation of the Varying IL Components

Phosphatidylethanolamine (PE, #840027) and phosphatidylglycerol (PG, #841188) extracted from *E. coli* were purchased from Avanti Polar Lipids (Alabama, USA). CHCl_3_ and phosphate buffer saline (PBS) tablets were provided by Sigma-Aldrich (Madrid, Spain). Ultrapure MilliQ water with a resistivity of 18.2 MΩ/cm was used in cleaning procedures and for PBS at pH 7.4 preparation. Gram-negative *E. coli* K12 NCTC 11047 was purchased from American Type Culture Collection (ATCC) (LGC Standards S.L.U) and used in all microbial assays. For the cytotoxicity tests, MC3T3-E1 preosteoblast cells were obtained from Riken Bank.

### 2.2 Ionic Liquid-Containing Polymeric Films

ILs with higher antimicrobial activity and/or membrane-disturbing capacity, namely, EMIM][HSO_4_], [DMIM][TFSI], and [P_6,6,6,14_][TFSI], were incorporated in a polymer matrix composed of poly(vinylidene fluoride-trifluoroethylene) (PVDF-TrFE). This was performed to assess the antimicrobial properties of the ILs when embedded in a polymeric film. Films with 20% (w/w_polymer_) IL content were developed according to ref (26). Briefly, pristine P(VDF-TrFE) films and IL-containing films were prepared in N,N-dimethylformamide (DMF) in a weight ratio of 15/85 (P(VDF-TrFE)/DMF). In the case of composite films, the ILs were first dispersed in DMF for 1 h with a magnetic stirrer. After complete IL dispersion, P(VDF-TrFE) was added to the solution and allowed to dissolve for 1 h. The prepared solution was then poured into a glass substrate and uniformly spread with an extender (doctor blade method) and placed at 210 °C for 10 min, allowing for rapid evaporation of the solvent and polymer melting. The morphology of the composites was further visualized by scanning electron microscopy (SEM) using a NOVA Nano SEM 200 FEI equipment operated at 10 kV. To obtain the image of the cross section, films were immersed in liquid nitrogen for a minute and broken into two pieces with the help of two clamps. The samples were then coated with a gold/palladium layer (approximately 10 nm thick) using a high-resolution sputter coater (208HR, Cressington).

### 2.3 Antibacterial Activity

For the antibacterial assays, the preparation of bacterial preinoculum was performed by incubating overnight at 37 °C at 110 rpm an isolated *E. coli* colony in nutrient broth (NB). Two methods have been used to investigate the antibacterial activity of ILs. The zone of inhibition test was performed to access the potential of ILs to leach and migrate in the agar, thus measuring the ability to kill bacteria by contact with solid surfaces, while the bacterial growth inhibition assay was performed in solution, thus mimicking aqueous environments. After 16 h, the *E. coli* concentration was adjusted to approximately 1 × 10^8^ colony forming units (CFUs) per ml for the zone of inhibition (ZoI) tests and to 1 × 10^6^ CFU/mL for the study of bacterial growth inhibition at different concentrations.

#### 2.3.1 Zone of Inhibition Assay

The procedure used to determine the ZoI was similar to that described by Padrão et al.^27^ Briefly, sterile 5 mm diameter Whatman 1 filter papers were immersed in each IL until saturation. Thereafter, the samples were placed on nutrient agar (NA) previously inoculated with *E. coli* in 90 mm diameter Petri dishes. The ZoI was determined after 12 h of incubation at 37 °C.

#### 2.3.2 Bacterial Growth Inhibition Assay

Bacterium susceptibility to the different ILs was assessed using the minimum inhibitory concentration (MIC) method.^28^ For this assay, different concentrations of ILs were prepared by serial dilution of the 50% (v/v) stock solution. The growth of bacteria was monitored using a microplate reader to measure the optical density (OD) at 600 nm, corrected for the absorbance of the corresponding blanks, i.e., the absorbance of the ionic liquids without any bacteria, after 24 h after incubating at 37 °C. The MIC was determined as the lowest concentration of IL at which no visible growth of *E. coli* was present, i.e., Abs_600_ = 0. The concentration in μmol/mL was calculated using the molecular weight of each IL.

#### 2.3.3 Colony Forming Units (CFUs) in Contact with Polymeric Films

The bactericidal activity of the IL-containing polymer films was assessed based on the standard ASTM-E2149-01 (shake flask method). This method provides quantitative data for measuring the reduction rate in the number of bacteria colonies formed, which were then converted to the average colony forming units per milliliter of buffer solution in the flask (CFU/mL). To evaluate the potential of the IL-containing polymer matrix to kill *E. coli*, samples 1 cm ×1 cm in size, previously sterilized under UV light for 30 min on each side, were placed in contact with each bacterial inoculum (1 mL of working inoculum) in 15 mL falcon tubes. The tubes were then subjected to vigorous agitation (220 rpm) at 37 °C for 2 h. The bacterial solution in contact with the material and respective controls was then removed, and the surviving colonies were quantified by serially diluting (1:10) in sterile buffer solution, plated on a plate count NB agar, and further incubated at 37 °C for 24 h. Antimicrobial activity is reported in terms of bacteria log_10_ reduction calculated as the ratio between the number of surviving bacteria after and before the contact with the materials, according to eq 1
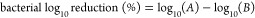
1where *A* and *B* are the average number of bacteria before and after contact with the samples, respectively. The results were further expressed as log_10_ reduction when compared to the control sample. All antibacterial data represent mean values of three independent assays ± SD (*n* = 3).

### 2.4 Cytotoxicity

A serial dilution of the 50% (v/v) IL stock solution was prepared and further tested for its biocompatibility. The ILs were then placed in contact with osteoblasts in Dulbecco’s modified Eagle medium (DMEM) in a 96-well tissue culture polystyrene plate for 24 h at 37 °C in 95% humidified air containing 5% CO_2_. Then, the leachable solution was analyzed using the 3-(4,5-dimethylthiazol2-yl)-2,5-diphenyltetrazolium bromide (MTT) assay method. This method evaluates cell viability, measuring the mitochondrial activity of cells, which is an indirect assessment of the number of viable cells. Briefly, MC3T3-E1 preosteoblast cells were seeded at a density of 2 × 10^4^ cells/mL in a 96-well tissue culture polystyrene plate and further incubated for 24 h. The culture medium was removed from the plate and replaced with the extraction medium that was previously in contact with ILs for 24 h. As a positive control, dimethyl sulfoxide (DMSO, Sigma-Aldrich) at a concentration of 20% (v/v) in DMEM was used, while as a negative control, the cell culture medium was used. After 24 h, the medium of every well was removed and 100 μL of MTT solution at a concentration of 10% (v/v) in DMEM (stock solution of 5 mg/mL MTT in PBS) was added to each well. Viable cells convert MTT into a purple formazan product, which, after 2 h of incubation, may be measured by dissolving the formed MTT crystals with DMSO. The OD was further measured at 570 nm using a spectrophotometric plate reader (Biotech Synergy HT). All quantitative results were obtained from five replicate samples and controls and analyzed as the average of cell viability ± standard deviation (SD), according to eq 2
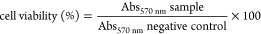
2

### 2.5 Ionic Liquid Mode of Action

#### 2.5.1 Langmuir Monolayer Formation

A PE and PG mixture was prepared at an 8:2 (v:v) ratio^29^ in CHCl_3_ (0.5 mg/mL). The Langmuir films were formed in a Langmuir trough equipped with two mobile barriers (KSV NIMA, model KN2002, Finland) with a total area of 273 cm^2^ mounted on an antivibration table and housed in an insulation box at 24 ± 1 °C. The Langmuir trough was cleaned with CHCl_3_ and water, and after subphase (PBS) addition, the surface was further cleaned by suction. Immediately, 40 μL of the lipid mixture solution was added dropwise into the trough, and after 10 min of evaporation of CHCl_3_, the barriers were compressed at 15 cm^2^/min until 33 mN/m, the equivalent of the natural membranes’ lateral surface pressure.^30^ After the stabilization of the membrane at the required surface pressure, at least 30 min, 100 μL of the corresponding ionic liquid diluted in 900 μL of PBS was inserted beneath the Langmuir film, and the changes of the surface pressure derived from the IL–membrane interactions were recorded. Blank experiments were carried out using the same procedure but 1000 μL of PBS for insertion.

#### 2.5.2 Membrane Permeabilization Assay

The extent of IL-induced membrane permeabilization was determined by measuring the release of cytoplasmic β-galactosidase from *E. coli* into the culture medium using ortho-nitrophenyl-β-galactoside (ONPG) as the substrate. Briefly, *E. coli* inoculum was harvested, washed, and resuspended in 0.9% NaCl solution. The final cell suspension was adjusted to obtain an OD of 1 at 420 nm. ILs (100 μL) were mixed with bacterial suspension (100 μL) in a 96-well plate and 30 mM ONPG acetone solution (10 μL). The production of *ο*-nitrophenol over time was monitored by the increase in absorbance at 420 nm using a spectrophotometer (TECAN Infinite M200, Austria).

## 3 Results and Discussion

### 3.1 Antibacterial Properties

**Figure 1:**
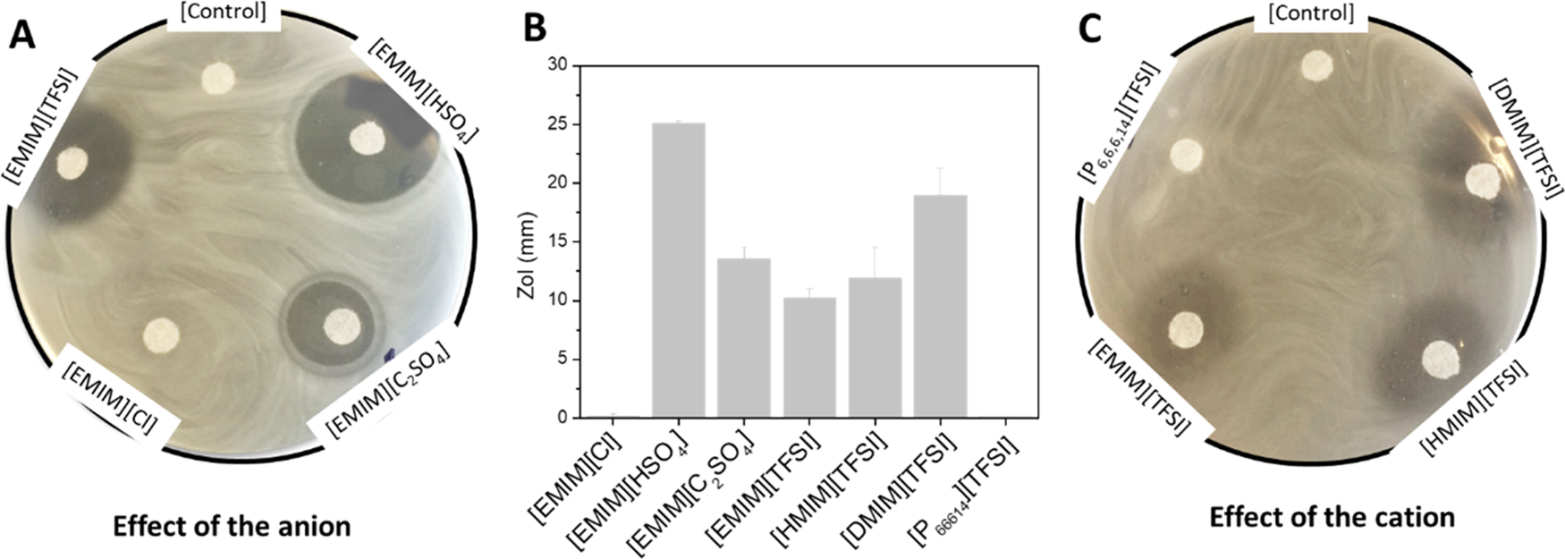
Zone of inhibition (ZoI) of ILs in contact with *E. coli*. (A) Agar plate denoting the effect of changing the anion, (B) quantitative measurement of all ZoI, and (C) agar plate denoting the effect of changing the anion.

ILs with different cations and anions have been selected to study the effect of their conjugation on the antibacterial activity and mode of action toward *E. coli*. Namely, ILs comprised of a specific cation [EMIM]^+^, with different anions, and ILs with a specific anion [TFSI]^−^, with different cations (Table 1), have been studied. Figure 1 represents the ZoI created by small filter paper disks impregnated with the ILs on an agar plate previously swabbed with *E. coli*. The results demonstrate the IL’s capacity to gradually release and migrate in the solid medium and kill this bacterium. In short, it is conventional to consider weak antimicrobial activity when the ZoI is lower than 12 mm, moderate activity when the ZoI is between > 12 and <20 mm, and strong antimicrobial activity when the ZoI is higher than 20 mm.^31^ According to the ZoI, [EMIM][HSO_4_] was found to be the most potent among the tested ILs, exhibiting a strong bactericidal activity and creating the largest clear halo on a plate of approximately 25 mm (Figure 1A,B). On the other hand, [P_66614_][TFSI] and [EMIM][Cl] were not able to migrate in the agar and inhibit the *E. coli* growth, showing the same effect as the control filter paper impregnated in PBS (Figure 1). The second largest ZoI, of approximately 18 mm, belongs to [DMIM][TFSI]. The ILs [EMIM][C_2_SO_4_] and [HMIM][TFSI] showed moderate bactericidal activity, presenting a ZoI of approximately 13 and 12 mm, respectively. [EMIM][TFSI] denotes a nearly moderate activity with a ZoI of 10 mm. Interestingly, [EMIM][C_2_SO_4_] and [EMIM][HSO_4_] display a clear border of their ZoI while the remaining borders are blurry. It is worth mentioning that the ILs comprising the [EMIM]^+^ cation show a higher antibacterial effect (Figure 1A), in agreement with previous results on the evaluation of the antimicrobial properties of this cation.^32−34^ Nevertheless, the fact that [EMIM][Cl] did not show any leachable bactericidal effect against *E. coli* indicates the importance of the anion on the capability of ILs to kill bacteria.

The ZoI test indicates the possibility of IL to leach and migrate to the agar, thus measuring its ability to kill bacteria in solid substrates by contact killing. This does not mean that the compounds with no ZoI have no antimicrobial activity. They may just not have the capacity to leach and act on the bacteria. The bacterial growth inhibition, on the other hand, is performed in an aqueous-based saline environment, thus showing the possibility of ILs killing bacteria in solution. Thus, the study of their bacterial growth inhibition at different concentrations was important to provide further insights into their efficacy and potential applicability. This is important information when considering biological applications of the ILs where the minimum concentration is usually required due to potential secondary or event toxicity effects toward mammalian cells.

**Figure 2:**
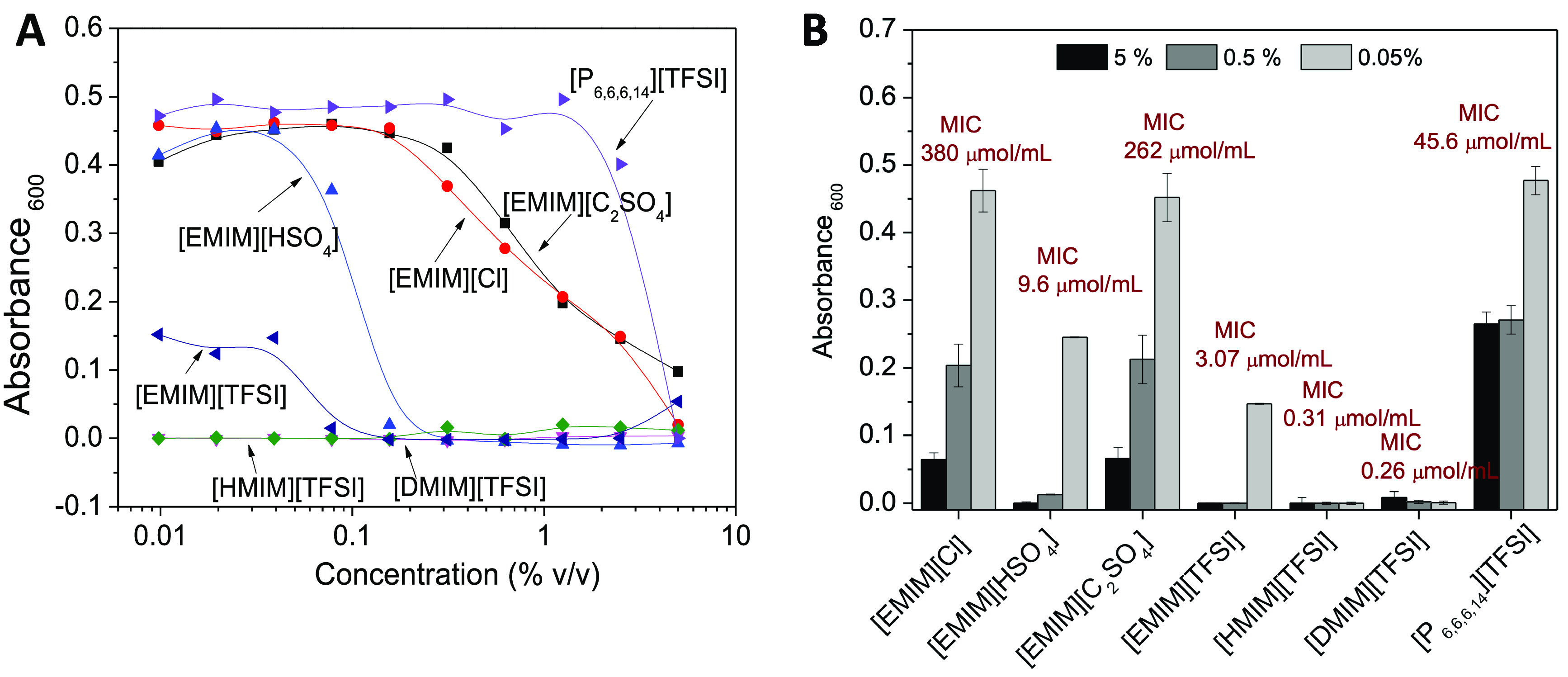
(A) Concentration-dependent antimicrobial activity (the *X*-axis is presented in log_10_ scale) and (B) details of the inhibition of *E. coli* in contact with 5%, 0.5%, and 0.05% v/v ILs and corresponding MICs calculated in μmol/mL.

As observed in Figure 2A,B, at a concentration of 5% v/v, all of the ILs show ability to inhibit *E. coli* growth to a certain extent. The trend then slowly changes when decreasing the concentration of ILs. The ILs [P_6,6,6,14_][TFSI], [EMIM][C_2_SO_4_], and [EMIM][Cl] in contact with *E. coli* rapidly lose their antimicrobial activity upon decreasing their concentration, showing higher MICs of 45.6, 262, and 380 μmol/mL, respectively (Figure 2A,B). The remaining ILs comprising the imidazolium moiety, [EMIM]^+^, [HMIM]^+^, and [DMIM]^+^ and the anion [TFSI]^−^, the ones with the largest antibacterial effects, show a remarkable inhibition trend even at low doses, showing bacterial inhibition with a decreasing value as the alkyl chain of the imidazolium cation increases and the effect being more pronounced with [DMIM]^+^. The MICs of these ILs were determined to be 3.07 μmol/mL for [EMIM]^+^, 0.31 μmol/mL for [HMIM]^+^, and 0.26 μmol/mL for [DMIM]^+^. These values are in good agreement with those of other imidazolium-based ILs, which have been reported to possess MIC ranging from 945 μmol/mL for 1-ethyl-3-vinylimidazolium bromide to 0.061 μmol/mL for 1-dodecyl-3-vinylimidazolium bromide, drastically decreasing with the increase in the length of the alkyl chain.^18^ These works emphasize the importance of the cation side chain for an improved antimicrobial outcome. Indeed, by fixing the anion [TFSI]^−^, different effects were observed, mainly if the length of the imidazolium alkyl chain is changed. The longer the alkyl chain, the better the inhibition effect. Cation groups with long side chains are reported to form a spatial heterogeneous region, aggregating in negatively charged microorganism membranes.^35^ Moreover, by changing the cation to a completely different one such as [P_6,6,6,14_]^+^, the antimicrobial activity is less potent, showing that the imidazolium moiety is more reactive toward bacteria than [P_6,6,6,14_]^+^ and inducing high antimicrobial effects when combined with the [TFSI]^−^ anion.

Imidazolium moieties have been reported to be responsible for the IL’s antimicrobial activity, but herein, the results indicate that the presence of different anions is also relevant for their antibacterial effect. [EMIM][HSO_4_] has shown a noteworthy MIC of 9.6 μmol/mL in comparison to the other anions combined with [EMIM], such as [C_2_SO_4_]^−^ and [Cl]^−^, which were found to be less effective combinations. Compounds comprising positive charges have long been shown to induce potent electrostatic interactions with the bacterial membrane due to their well-established negative charges due to the presence of phosphate groups on the cell wall. The longer the alkyl chains, the easier the interactions, inducing cell lysis and death.

### 3.2 Assessment of the IL Mode of Action on E. coli

**Figure 3:**
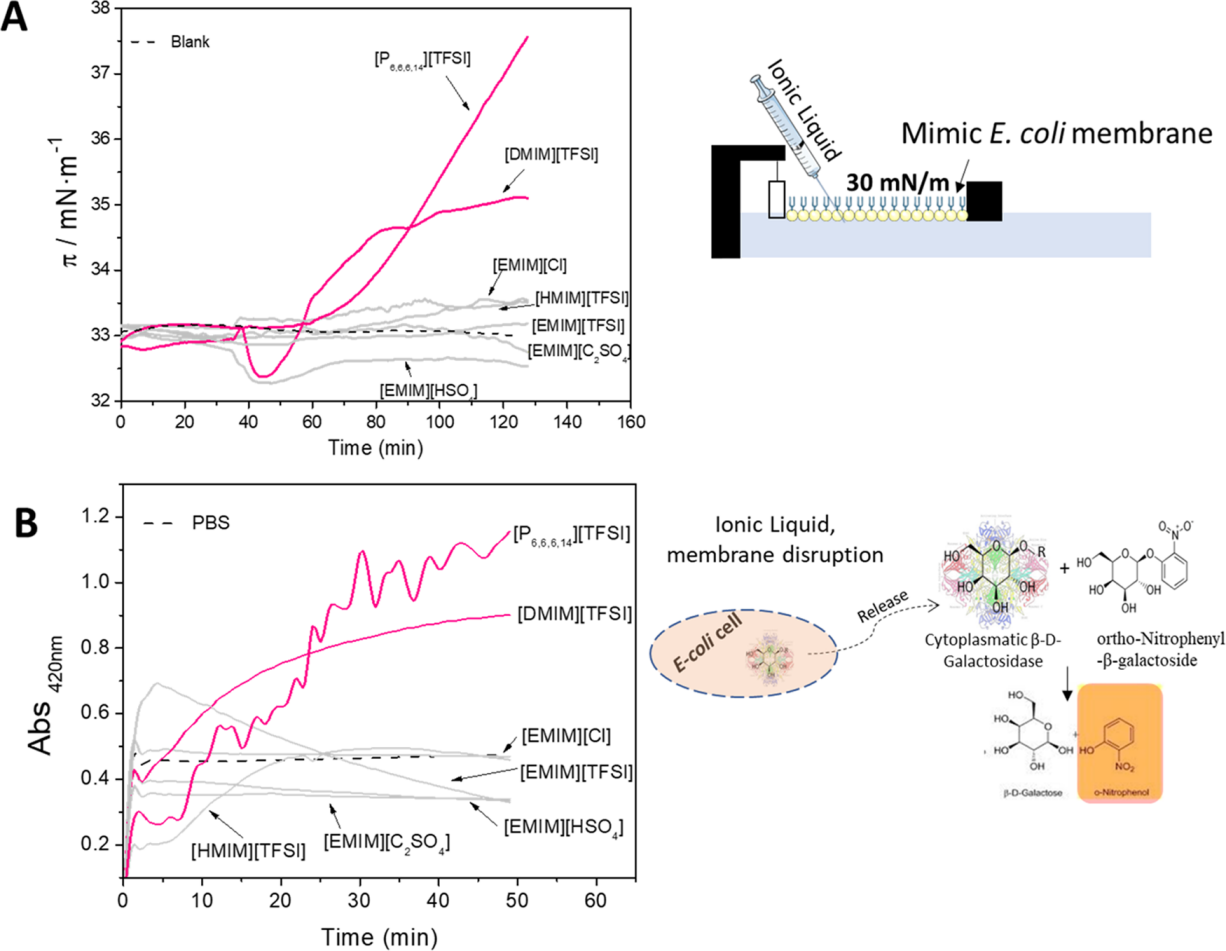
(A) Kinetic adsorption resulting from the incorporation of ILs (0.05% v/v) (final concentration) into the air–water interface of *E. coli* PE:PG monolayer at a surface pressure of 33 mN/m and schematic representation of the incorporation of the ILs in the subphase beneath the phospholipid monolayer. (B) Release of cytoplasmic β-galactosidase from the *E. coli* cells in contact with the ILs at 0.05% v/V and the control (PBS) and respective schematic representation of the enzyme release and its further detection using ONPG.

The IL mode of action toward *E. coli* was evaluated through its capacity to induce an effective interaction with a bacterial membrane model or through the lysis of bacterial cells. The membrane model comprised of a phospholipid mixture of PE:PG in an 8:2 ratio, the main phospholipids extracted from *E. coli* membranes,^29^ was assembled in the water–air interface using a Langmuir monolayer technique (Figure 3A), and the ILs were placed in the water subphase using a syringe. This technique has been commonly applied to elucidate the interactions of antimicrobial agents with bacterial cell membranes at a molecular level, thus indirectly assessing their antibacterial potential.^36−38^ In fact, it has been widely used for building monolayers that mimic natural membranes and provides information about the interactions between structural lipids and different molecules or nanoparticulate systems, without considering other constituents of the cell membrane.^39−41^

The *E. coli* PE:PG monolayer was formed at 33 mN/m to mimic the surface pressure of a bacterial cell membrane, and the ILs were further injected into the subphase beneath. The variation in the surface tension of the monolayer was then monitored as a function of time (Figure 3A). The [DMIM][TFSI] and [P_6,6,6,14_][TFSI] ILs were found to efficiently penetrate the membrane model, as indicated by the higher increase of the surface pressure in comparison to the other ILs. These results show that these ILs are surface-active and strongly interact with the membrane model and, therefore, with the bacterial membrane. The other ILs had no measurable effect on the membrane model.

The bacterial membrane of *E. coli*, as a Gram-negative bacteria, possesses a lipidic membrane and an outer bacterial wall made of lipopolysaccharides, making it more difficult for the ILs to penetrate the membrane. Thus, the measurement of the leakage of intracellular material from *E. coli* was performed to corroborate the Langmuir monolayer results, as this only mimics the outer membrane of *E. coli*. The evaluation of the *E. coli* inner membrane permeabilization as a function of cytoplasmic β-galactosidase release was also studied. When *E. coli* inoculum was incubated with the ILs, an immediate release of β-galactosidase was observed for the bacteria incubated with [DMIM][TFSI] and [P_6,6,6,14_][TFSI] (Figure 3B), and the same ILs were shown to disturb the monolayer in the Langmuir technique experiments. The other ILs induced a negligible effect, not inducing enzyme release, which suggests the lack of efficacy in disrupting the cell membrane. Since all ILs possess antibacterial activity to a certain extent (Figure 2), the mechanism of action of the ILs with the most potent antibacterial activity, namely, [EMIM][TFSI], [HMIM][TFSI], and [EMIM][HSO_4_], is certainly based on the chemical phenomenon (e.g., by blocking vital processes in bacteria similar to the way in which most antibiotics act) rather than on physical ones (e.g., cell membrane disruption).

The fact that [P_6,6,6,14_][TFSI] is the most surface-active IL, but possesses less potent antimicrobial activity when compared with other ILs, indicates that the membrane-disruption capacity may be only effective at higher concentrations. In fact, being surface-active does not necessarily mean that it should have a potent antimicrobial effect. It rather indicates that the mechanism of action is associated with bacterial cell membrane disruption, which is very important for us to infer that it avoids the occurrence of antimicrobial resistance.^36−38^ In the case of [DMIM][TFSI], the IL is effective in penetrating the membrane and inducing a strong bacterial growth inhibition, thus making this IL a good antimicrobial agent. Both membrane-active ILs have one feature in common: the long alkyl chains on the cation. As previously explained and widely reported, the long alkyl chains aggregate and interact more easily with the bacteria.^18^

ILs possess similar chemical structures to those of other well-established cationic biocides and surfactants, possessing characteristics such as a charged hydrophilic head group and hydrophobic ‘tails’.^42^ Such similarities indicate that ILs may aggregate in solution to form amphiphilic micelles,^42,43^ whose capacity increases with increasing lipophilicity, which in turn may be manipulated by extending the substituent alkyl chain. Micelles are able to disrupt the integrity of the membrane through electrostatic interaction between the cationic groups of the ILs and the anionic groups of the cell membrane, resulting in the loss of the barrier function of the outer membrane.^44^ This is the most widely accepted mechanism of action of ILs toward microorganisms such as bacteria, which is mainly attributed to the IL cation. In fact, many physical phenomena of aggregation of different types of ILs with different cations at the surface of bacterial cells such as *E. coli* have been reported.^11,45^

**Figure 4:**
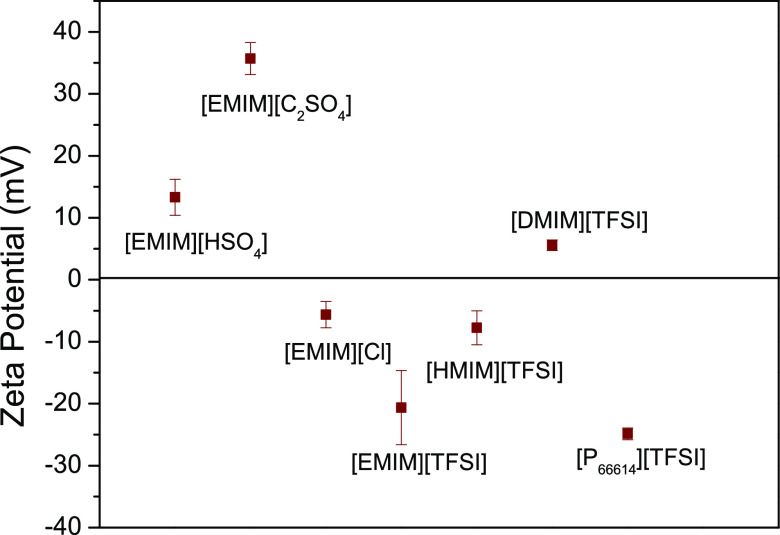
IL ζ potential, which is indicative of the overall charge of these compounds, measured at a physiological pH of 7.4.

Therefore, the ζ potential of the ILs at physiologic pH was analyzed to infer possible electrostatic interactions that may occur between the ILs and the bacterial membrane. As shown in Figure 4, among the ILs with bacterial cell-disrupting capacity, only [DMIM][TFSI] is positively charged, which could explain its antibacterial activity against *E. coli*. On the other hand, [P_6,6,6,14_][TFSI] is negatively charged, which in combination with the information shown in Figure 3 about the effective interaction with the bacterial membrane suggests that it interacts with the membrane through nonspecific binding and clustering on the cationic sites of the membrane. Similar observations have been reported in ref (38) where negatively charged penicillin nanoparticles were able to eradicate otherwise nonsusceptible Gram-negative bacteria through effective membrane interaction. Indeed, bioengineering simulation studies of the interaction of ILs at membrane model interface have demonstrated that either cations or anions are able to insert themselves into a lipid bilayer, changing the structural and dynamic properties of the bilayer and leading to their permeability.^46^

### 3.3 In Vitro Cytotoxicity with Mammalian Cells

**Figure 5:**
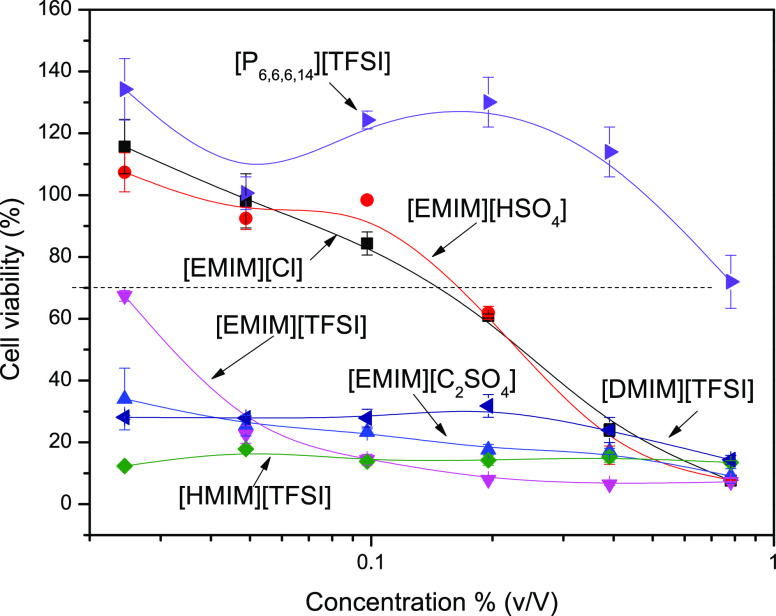
Dose-dependence cell viability of ILs in contact with preosteoblast for 24 h. The *X*-axis is presented in a log_10_ scale.

Cell culture-based assays were used as a prescreening tool to understand the biological effects of the different compounds on human cells. This feature is important to assess since the herein studied ILs possess high antibacterial activity and could be potentially harmful to human cells as well. The potential cytotoxicity of the ILs was thus assessed using the cell viability reduction method with a human preosteoblastic cell line (Figure 5).

The [EMIM][HSO_4_] and [EMIM][Cl] ILs are found to be nontoxic at concentrations below approximately 0.5% (v/v), while [P_6,6,6,14_][TFSI] IL is nontoxic up to approximately 1% (v/v). All other ILs were found to be cytotoxic. These results are particularly interesting since [EMIM][HSO_4_] is highly antimicrobial toward *E. coli* at 0.5% (v/v). On the other hand, [EMIM][Cl] is also antimicrobial at a safe concentration of 0.5% (v/v). These ILs are both biocompatible and nontoxic, which makes them suitable for a wide range of biomedical applications, including for coating indwelling medical devices with antimicrobial moieties^47,48^ and for material processing intended for topical delivery of anesthetic and anti-inflammatory drugs.^49,50^ [P_6,6,6,14_][TFSI] is also biocompatible at all concentrations tested but antimicrobial only at high concentrations, and this fact has to be taken into consideration when specific biomedical applications are considered.

### 3.4 Antimicrobial Properties of the IL-Containing Polymer Films

**Figure 6:**
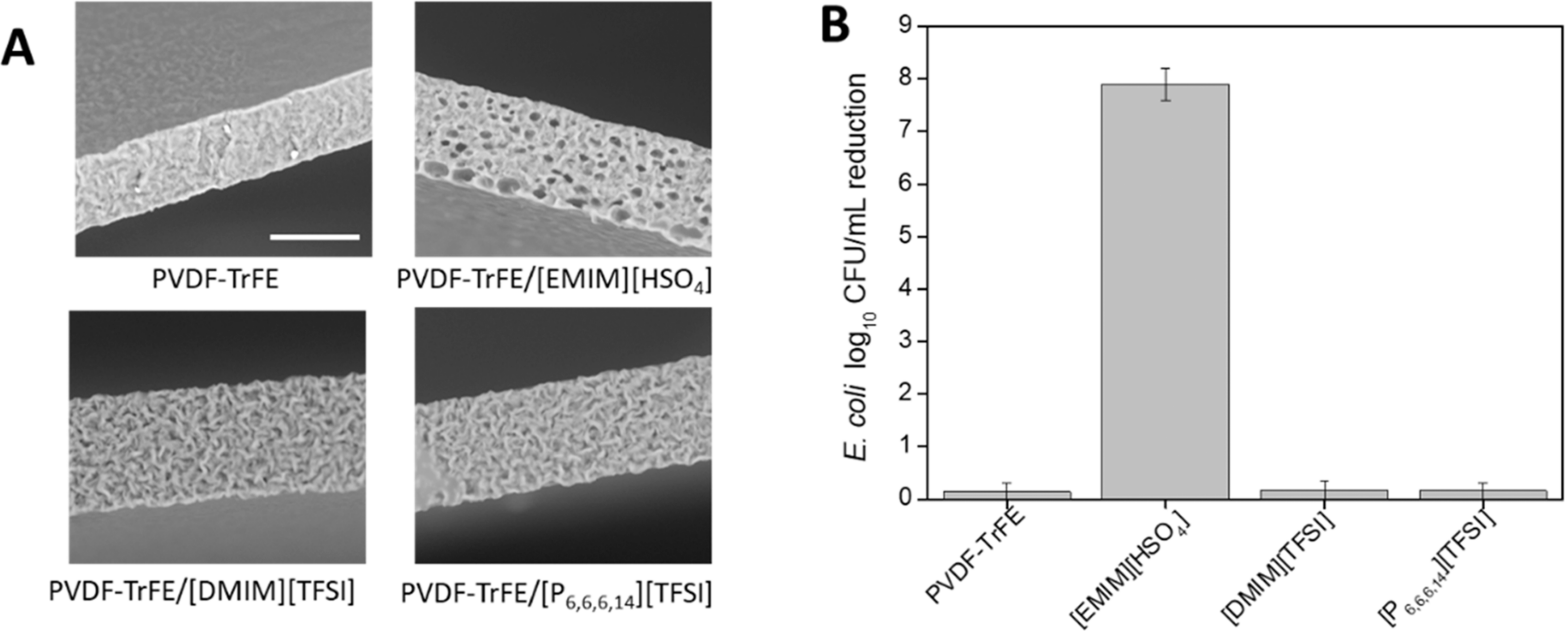
IL-containing polymeric (PVDF-TrFE) films. (A) Cross section by SEM (bar represents 30 μm) and (B) antimicrobial activity against *E. coli*.

Based on the antimicrobial properties of the ILs and/or their membrane-disturbing capacity, [EMIM][HSO_4_], [DMIM][TFSI], and [P_6,6,6,14_][TFSI] were selected to be incorporated in a polymer matrix composed of PVDF-TrFE. The films were obtained using a simple solvent-casting method to evaluate the possibility of creating an antimicrobial material. From an application point of view, the creation of films and/or membranes with high antimicrobial properties is a prerequisite for biomedical applications. The only material found to be antimicrobial was the one comprising [EMIM][HSO_4_], inducing an impressive CFUs log_10_ reduction of 7 (Figure 6B). The other materials did not possess the capacity to kill *E. coli*, possessing a bacterial CFU reduction of around 0.5, the same as that of the pristine PVDF-TrFE film. From the cross-sectional SEM images depicted in Figure 6A, it can be seen that [EMIM][HSO_4_] induces the formation of pores on the material, which might be an important feature for improved contact of the material with the bacteria.

## 4 Conclusions

Exploring different antimicrobial agents that are not commonly used for such purposes constitutes a powerful tool for tackling the resistance of Gram-negative bacteria such as *E. coli* to antibiotics. This bacterium is particularly important since it is an opportunistic pathogen present in clinical settings, causing frequent nosocomial infections. In this work, the antibacterial activities of several ILs on *E. coli* were investigated. It was shown that the length of the alkyl chain linked to the imidazolium cation plays an important role in the IL antimicrobial activity inducing remarkably high antimicrobial activity even at a low concentration of 0.5% v/v for the [DMIM][TFSI] ionic liquid. This IL possesses a long alkyl chain, a feature shared by [P_6,6,6,14_][TFSI] and associated with its capacity to disturb the membrane of bacteria, proven by *in vitro* membrane model techniques such as the Langmuir monolayer technique. These ionic liquids are surface-active toward the membrane model and induce lysis of the bacterial cell wall membrane in a mechanism governed by effective electrostatic interactions. The antimicrobial capacity of the ILs was also evaluated in terms of their capacity to migrate to the medium and create an inhibition zone and through the determination of their potential to inhibit bacterial growth in solution.

[EMIM][HSO_4_] has shown high antimicrobial properties with a remarkably low MIC, biocompatibility, and leachable capacity, being the most suitable IL for biomedical applications. Besides these properties, it was able to induce antimicrobial properties in a polymeric material in the form of a film. This work has also shown that by varying the imidazolium-like cation to trihexyltetradecyl phosphonium [P_6,6,6,14_]^+^, a decrease in the antimicrobial capacity of the IL is observed, but its biocompatibility is improved, suggesting that imidazolium-like cations are responsible for the IL toxicity toward both human and bacterial cells.
